# Author Correction: Estimating countries’ additional carbon accountability for closing the mitigation gap based on past and future emissions

**DOI:** 10.1038/s41467-024-55438-w

**Published:** 2024-12-18

**Authors:** Thomas Hahn, Johannes Morfeldt, Robert Höglund, Mikael Karlsson, Ingo Fetzer

**Affiliations:** 1 Stockholm Resilience Centre, Stockholm University, Stockholm, Sweden; 2 Physical Resource Theory, Department of Space, Earth and Environment, Chalmers University of Technology, Gothenburg, Sweden; 3 Marginal Carbon AB, Stockholm, Sweden; 4 Climate Change Leadership, Department of Earth Sciences, Uppsala University, Uppsala, Sweden; 5 Bolin Centre for Climate Research, Stockholm University, Stockholm, Sweden

**Keywords:** Climate-change mitigation, Climate-change policy

Correction to: *Nature Communications* 10.1038/s41467-024-54039-x, published online 09 November 2024

The original version of this Article contained an error in sub-section “Estimating countries’ additional carbon accountability”, which incorrectly read ‘Four of 18 countries with additional carbon accountability have larger planned future emissions than additional accountabilities: China, Türkiye, Iran, and Thailand. They could theoretically fulfil their entire additional carbon accountability with stricter domestic emission reductions than stipulated in their NDCs and NZTs. For China, Iran and Thailand this would however require more than halving their planned future emissions, hence CDR is probably needed too.’ The correct version replaces this sentence with ‘Five of 18 countries with additional carbon accountability have larger planned future emissions than additional accountabilities: Türkiye, China, New Zealand, Iran and South Africa. They could theoretically fulfil their entire additional carbon accountability with stricter domestic emission reductions than stipulated in their NDCs and NZTs. For China, New Zealand, Iran and South Africa, this would however require a large portion of their planned future emissions, hence CDR is probably needed too’.

This has been corrected in both the PDF and HTML versions of the Article.

The original version of the Supplementary Information associated with this Article contains line numbers and highlighted revision. The HTML has been updated to include a corrected version of the Supplementary Information.

## Supplementary information

Revised Supplementary Information

