# Supplementary material for: Estimating countries’ additional carbon accountability for closing the mitigation gap based on past and future emissions
Source: Nat Commun. 2024 Nov 9;15:9707. doi: 10.1038/s41467-024-54039-x (PMC11550376; doi:10.1038/s41467-024-54039-x)
Supplement: Supplementary file 1 — Supplementary Information [file 41467_2024_54039_MOESM1_ESM.pdf]

Supplementary Information

Estimating countries' additional carbon accountability for closing the mitigation gap based on past and future emissions

Thomas Hahn[1], Johannes Morfeldt[2], Robert Höglund[3], Mikael Karlsson[4], Ingo Fetzer[1,5]

1. Stockholm Resilience Centre, Stockholm University, Albanovägen 28, SE-114 19 Stockholm
2. Physical Resource Theory, Department of Space, Earth and Environment, Chalmers University of Technology, Maskingränd 2, SE-412 96 Gothenburg, Sweden.
3. Marginal Carbon AB, Stockholm, Sweden.
4. Climate Change Leadership, Department of Earth Sciences, Uppsala University, Villavägen 16, SE-752 36 Uppsala, Sweden.
5. Bolin Centre for Climate Research, Stockholm University, Svante Arrhenius väg 8 C, SE-114 18 Stockholm, Sweden.

Table of Contents

Supplementary Tables **2**

Supplementary Figures **5**

Supplementary Notes **11**

Supplementary Note 1: Allocation methods used in the sensitivity analyses. **11**

Supplementary References **14**

Supplementary Tables

Supplementary Table 1. **Additional Carbon Accountability for achieving 2°C with 83% probability.** The table shows results for the analysed countries, their income-level, and GDP per capita. The results are shown for carbon debt (i.e., a country's emissions during 1990-2022 minus the country's equal per capita share in the global cumulative emissions over the same period), future carbon claims (i.e., cumulative planned emissions during 2023-2070 if the country achieves its NDC and NZT), excessive carbon claims (i.e., the country's cumulative planned emissions minus an equal per capita share of the remaining carbon budget for 2023-2070), total excessive carbon claims (i.e., the sum of the carbon debt and excessive carbon claims for the country), the additional carbon accountability (i.e., the responsibility for mitigation or removal of CO_2 additional to the country achieving its NDC and NZT), the additional carbon accountability per capita (based on the country's average population during 2023-2070).

Country	Income-level	Carbon Debt ($MtCO_2$)	Future Carbon Claims ($MtCO_2$)	Excessive carbon claim ($MtCO_2$)	Total excessive carbon claims ($MtCO_2$)	Additional Carbon Account-ability ($MtCO_2$)	Additional Carbon Account-ability per capita (tCO_2)
Norway	High	681	378	-134	547	0	0
Switzerland	High	259	548	-241	18	0	0
Singapore	High	874	856	342	1,216	0	0
United States	High	138,705	62,673	32,262	170,967	12,075	33
Australia	High	8,974	5,635	3,056	12,030	0	0
Canada	High	13,046	6,749	3,058	16,105	0	0
United Arab Emirates	High	3,848	5,338	4,421	8,270	3,481	313
New Zealand	High	474	435	-45	429	0	0
United Kingdom	High	7,590	4,128	-1,699	5,891	0	0
European Union	High	50,351	35,702	1,527	51,878	0	0
Japan	High	21,328	14,242	5,477	26,805	0	0
South Korea	High	9,510	8,490	4,708	14,218	0	0
Saudi Arabia	High	10,735	14,839	11,047	21,782	1,969	43
Russia	High	33,726	43,041	31,995	65,721	8,009	60
Chile	High	-319	1,365	-305	-624	0	0
Argentina	Upper-middle	-596	3,696	-451	-1,047	0	0
Costa Rica	Upper-middle	-429	123	-335	-764	0	0
China	Upper-middle	25,208	246,446	138,767	163,975	0	0
Kazakhstan	Upper-middle	4,767	5,728	3,690	8,457	0	0
Mexico	Upper-middle	-1,666	14,514	2,998	1,331	0	0
Türkiye	Upper-middle	-855	10,031	2,359	1,503	0	0
Brazil	Upper-middle	-14,973	7,615	-11,042	-26,015	0	0
Peru	Upper-middle	-2,978	1,065	-2,277	-5,255	0	0
Thailand	Upper-middle	-2,790	6,912	1,339	-1,450	0	0
South Africa	Upper-middle	6,132	6,248	403	6,534	0	0
Colombia	Upper-middle	-3,909	1,482	-3,103	-7,013	0	0
Indonesia	Upper-middle	-21,376	23,575	-1,842	-23,217	0	0
Iran	Upper-middle	4,823	32,300	24,387	29,210	0	0
Egypt	Lower-middle	-6,546	9,318	-3,253	-9,799	0	0
Viet Nam	Lower-middle	-8,131	7,304	-1,335	-9,466	0	0
Philippines	Lower-middle	-10,254	4,896	-7,551	-17,805	0	0
Morocco	Lower-middle	-3,098	2,237	-1,358	-4,456	0	0
India	Lower-middle	-123,182	92,093	-41,401	-164,584	0	0
Nigeria	Lower-middle	-18,970	3,036	-26,173	-45,143	0	0
Kenya	Lower-middle	-5,191	683	-5,948	-11,139	0	0
Ethiopia	Low	-11,919	307	-16,359	-28,278	0	0
Gambia	Low	-252	14	-347	-599	0	0
Rest of world	Rest of world	-103,596	116,492	-121,105	-224,701	0	0
World	World	0	800,534	25,534	25,534	25,534	3

Supplementary Table 2. **Assumptions for estimating countries' planned emissions.** The table shows assumptions made to quantify the planned emissions of each country, including the country's 2030 target as formulated in their NDC and the year for reaching net zero. Note that net zero years marked with an asterisk (*) indicate that the country lacks a net-zero target and that the country is assumed to reach net-zero by 2070.

Country	Quantification of 2030 target based on Climate Action Tracker	Net Zero Year
Argentina	16% above 2010	2050
Australia	25% below 2010	2050
Brazil	4% below 2010	2050
Canada	38% below 2010	2050
Chile	7% above 2010	2050
China	26% above 2010	2060
Colombia	8% above 2010	2050
Costa Rica	9% below 2010	2050
Egypt	72% above 2010	2070*
Ethiopia	99% above 2010	2060
European Union	52% below 1990	2050
Gambia	95% above 2010	2050
India	104% above 2010	2070
Indonesia	148% above 2010	2060
Iran	125% above 2010	2070*
Japan	38% below 2010	2050
Kazakhstan	1% above 2010	2060
Kenya	104% above 2010	2070*
Mexico	16% above 2010	2070*
Morocco	53% above 2010 levels	2070*
New Zealand	34% below 2010	2050
Nigeria	22% above 2010	2060
Norway	52% below 1990	2050
Peru	43% above 2010	2050
Philippines	119% above 2010	2070*
Russia	20% above 2010	2060
Saudi Arabia	25,5% above 2010	2060
Singapore	11% above 2010	2050
South Africa	25,5% below 2010	2050
South Korea	32% reduction below 2018	2050
Switzerland	31,515% below 1990	2050
Thailand	15% above 2010	2065
Türkiye	92% above 2010	2050
United Arab	1% above 2010	2070*
United Kingdom	58% below 2010	2050
United States	43,5% below 2010	2050
Viet Nam	212% above 2010	2050
Rest of world	Constant at 2022 level	2070*

Supplementary Figures

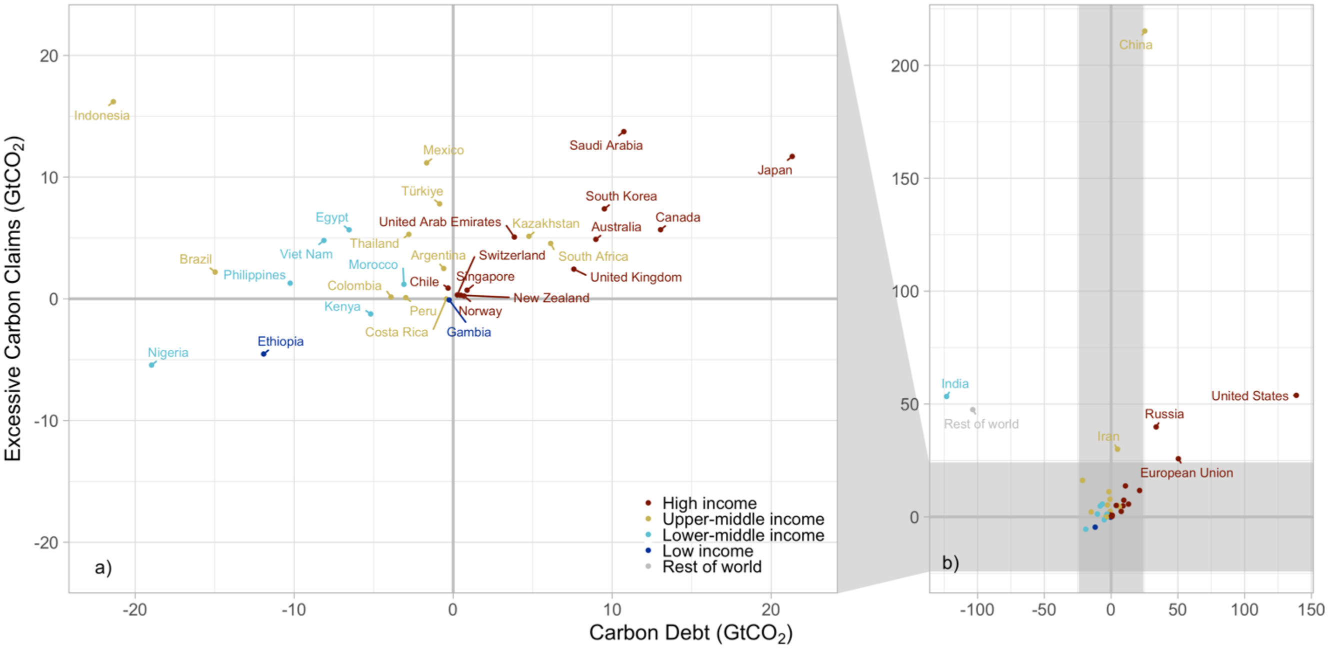

Supplementary Figure 1. **Excessive carbon claims and carbon debt for achieving 1.5°C budget at 50% probability**. The figure shows the excessive carbon claims during 2023-2070 (y-axis) and carbon debt during 1990-2022 (x-axis) for countries of different income-levels (color). The left panel (a) is an expansion of the right panel (b). Countries in the upper right quadrant have a positive carbon debt (i.e., higher emissions during the period than the cumulative per-capita allocation of the global cumulative emissions over the same period) and positive excessive carbon claims (i.e., their planned emissions exceed the cumulative per-capita allocation of the remaining carbon budget 2023-2070).

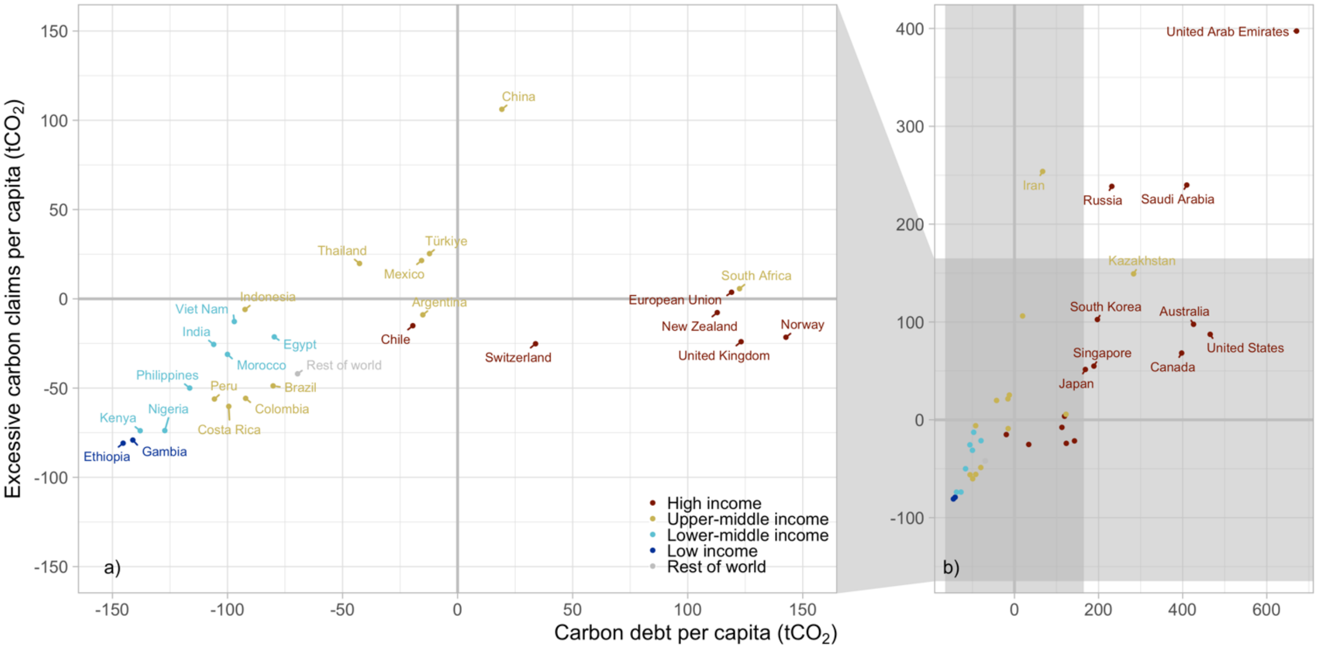

Supplementary Figure 2. **Excessive carbon claims and carbon debts per capita for achieving 2°C budget at 83% probability.** The figure shows the excessive carbon claims per capita during 2023-2070 (y-axis) and carbon debt per capita during 1990-2022 (x-axis) for countries of different income-levels (color), based on average populations during the analysed periods. The left panel (a) is an expansion of the right panel (b). Countries in the upper right quadrant have a positive carbon debt (i.e., higher emissions during the period than the cumulative per-capita allocation of the global cumulative emissions over the same period) and positive excessive carbon claims (i.e., their planned emissions exceed the cumulative per-capita allocation of the remaining carbon budget 2023-2070).

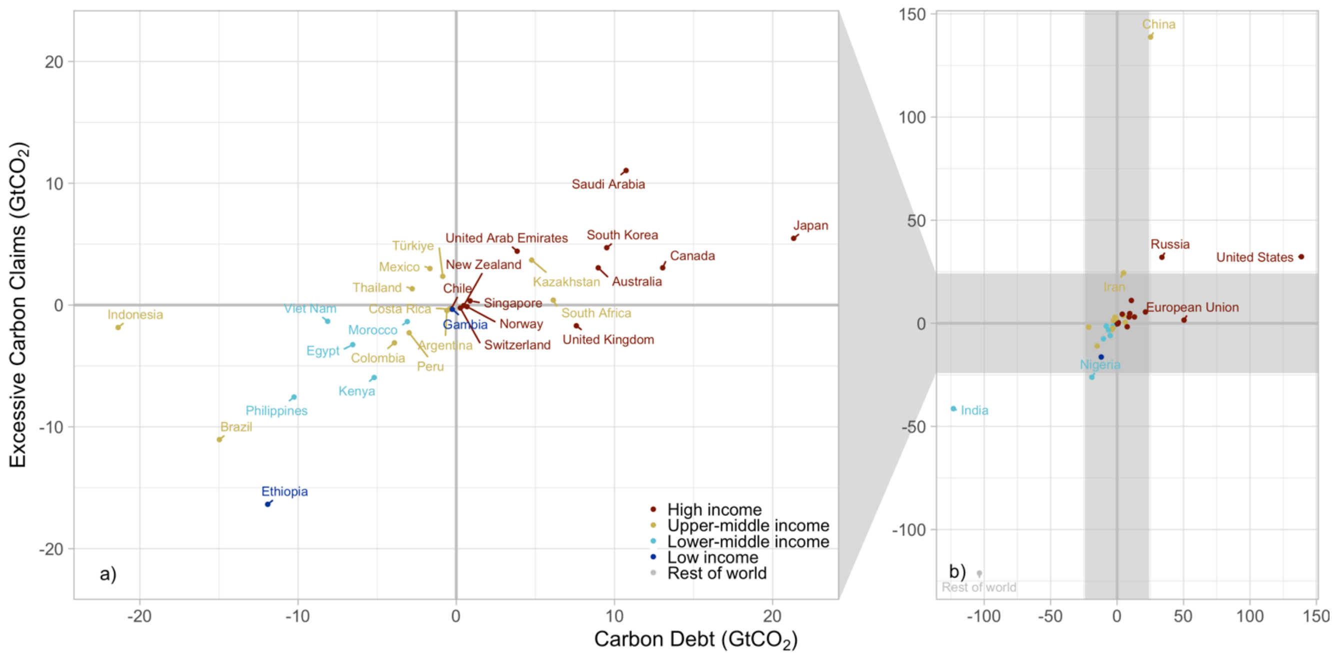

Supplementary Figure 3. **Excessive carbon claims and carbon debts for achieving 2°C budget at 83% probability.** The figure shows the excessive carbon claims during 2023-2070 (y-axis) and carbon debts during 1990-2022 (x-axis) for countries of different income-levels (color). The left panel (a) is an expansion of the right panel (b). Countries in the upper right quadrant have a positive carbon debt (i.e., higher emissions during the period than the cumulative per-capita allocation of the global cumulative emissions over the same period) and positive excessive carbon claims (i.e., their planned emissions exceed the cumulative per-capita allocation of the remaining carbon budget 2023-2070).

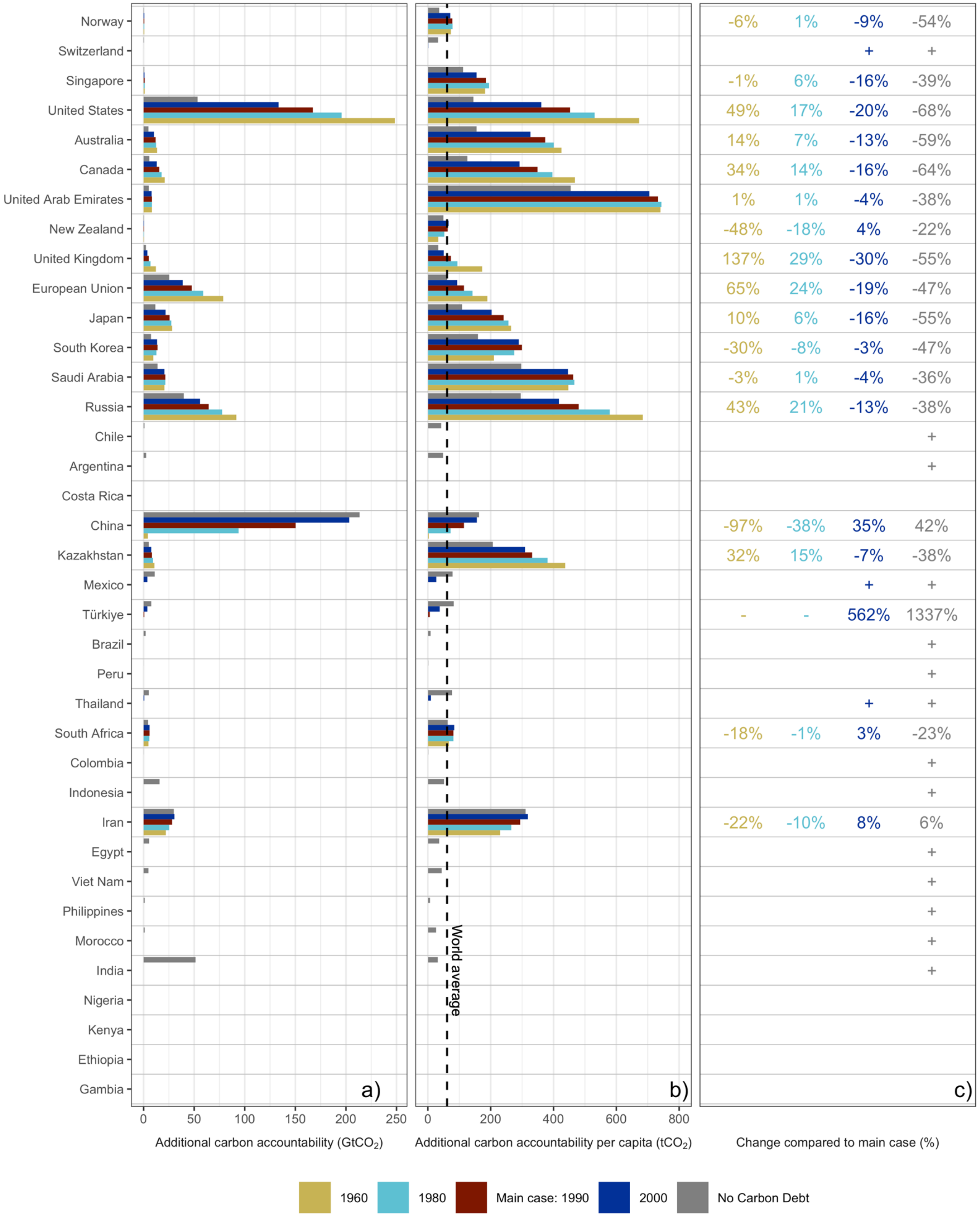

Supplementary Figure 4. **Sensitivity analysis of starting year for carbon debt calculations. a** Additional carbon accountability, **b** Additional carbon accountability per capita, and **c** change in additional carbon accountability as compared to the main case presented in the article, for different assumptions on the starting year for the carbon debt calculations – 1960 (beige), 1980 (turquoise), 1990 as in the main case (maroon), 2000 (blue), and no carbon debt (gray). The allocation of the remaining carbon budget and the redistribution of the emission allowances pool are based on the equal cumulative per capita principle for all cases. The results are for achieving a 1.5°C budget at 50% probability.

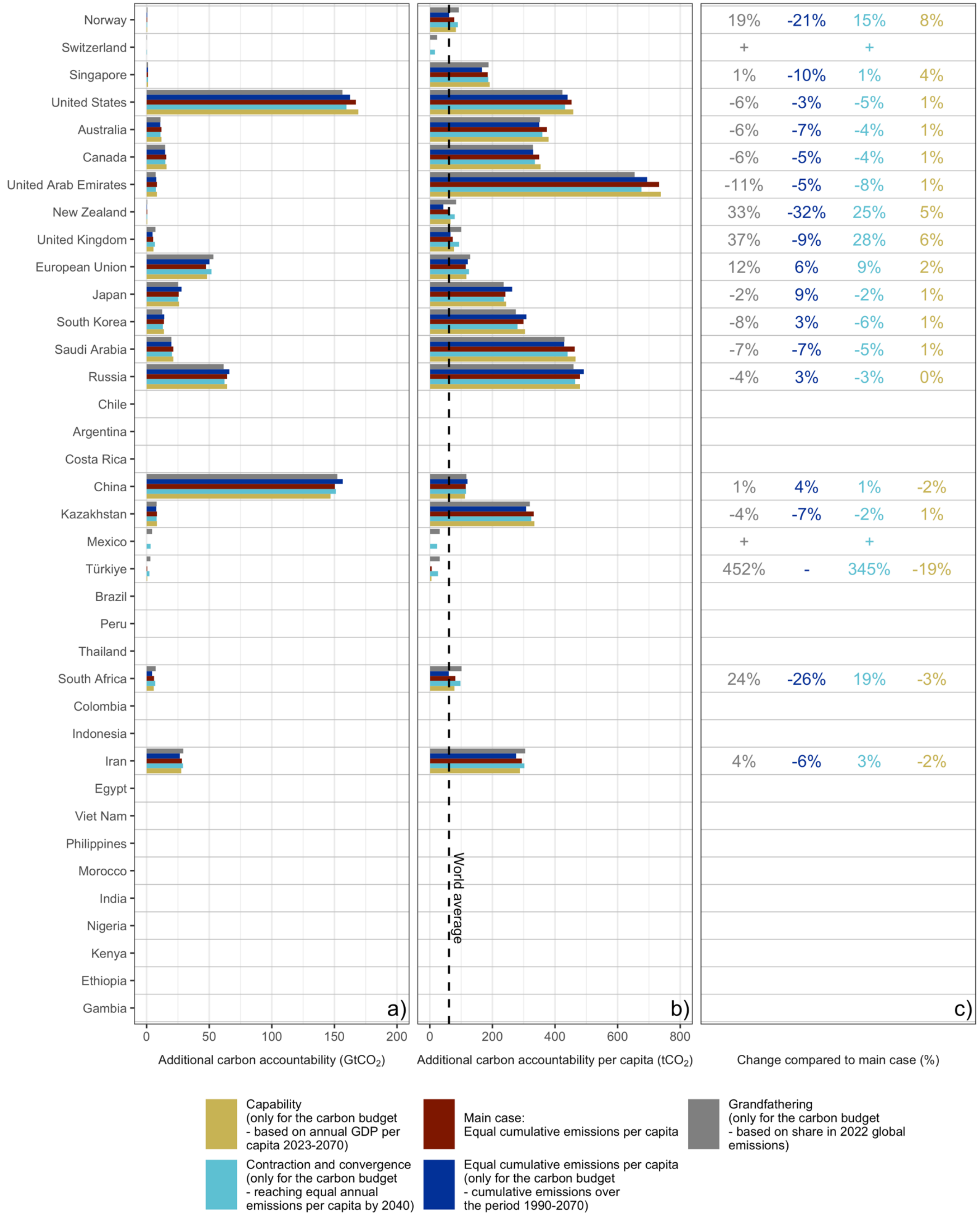

Supplementary Figure 5. **Sensitivity analysis of the chosen allocation principle for the carbon budget. a** Additional carbon accountability, **b** Additional carbon accountability per capita, and **c** change in additional carbon accountability as compared to the main case presented in the article, for different allocation principles – capability (mustard), contraction and convergence (turquoise), grandfathering (gray), main case (maroon), and equal cumulative emissions per capita from 2023 (blue). All cases assume responsibility for carbon debt since 1990 and the redistribution of the emission allowances pool is based on the equal cumulative per capita principle. The results are for achieving a 1.5°C budget at 50% probability.

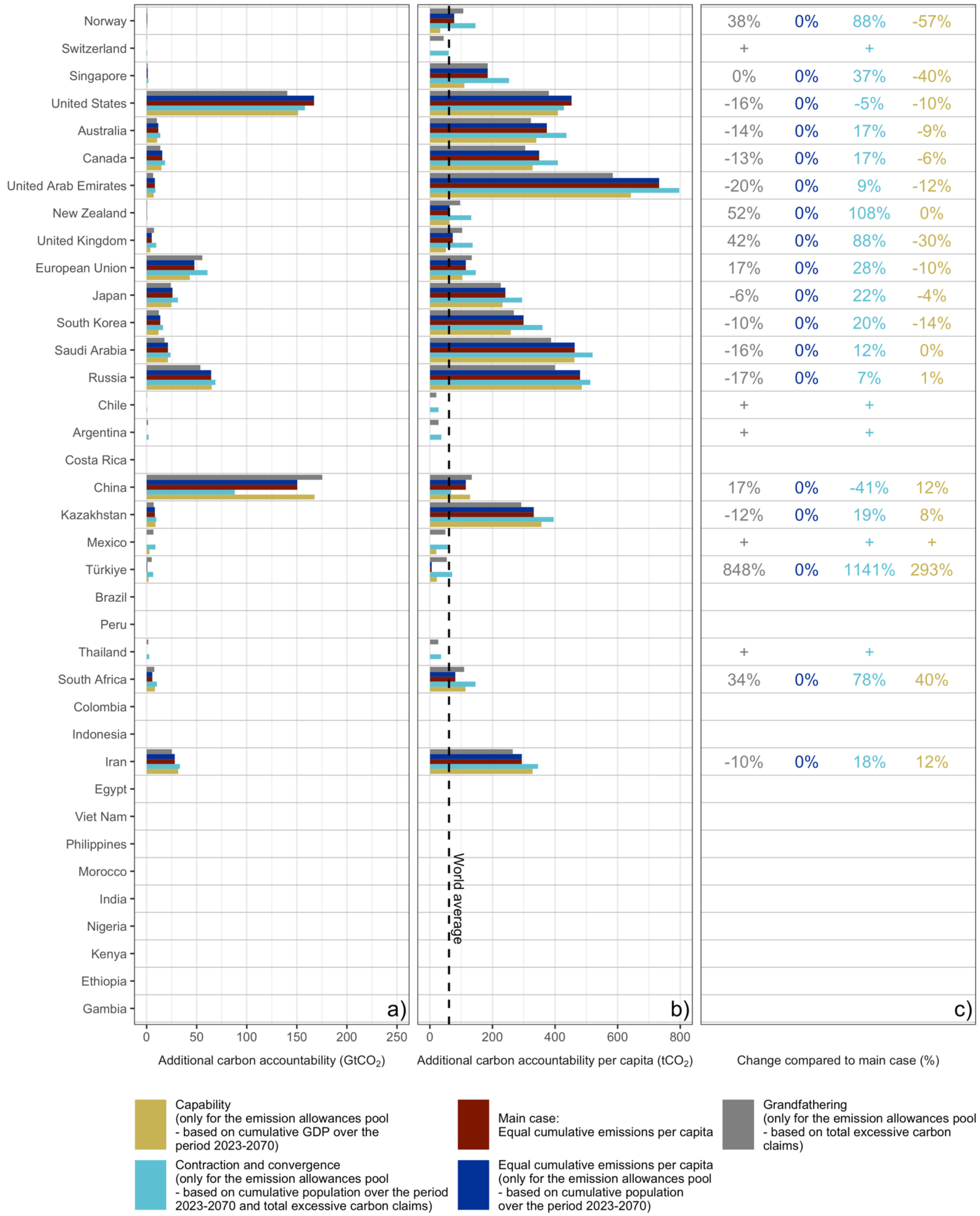

Supplementary Figure 6. **Sensitivity analysis of the chosen principle for redistributing the emission allowances pool. a** Additional carbon accountability, **b** Additional carbon accountability per capita, and **c** change in additional carbon accountability as compared to the main case presented in the article, for different allocation principles – capability (mustard), contraction and convergence (turquoise), grandfathering (gray), main case (maroon), and equal cumulative emissions per capita from 2023 (blue). All cases assume responsibility for carbon debt since 1990 and the allocation of the remaining carbon budget is based on the equal cumulative per capita principle. The results are for achieving a 1.5°C budget at 50% probability.

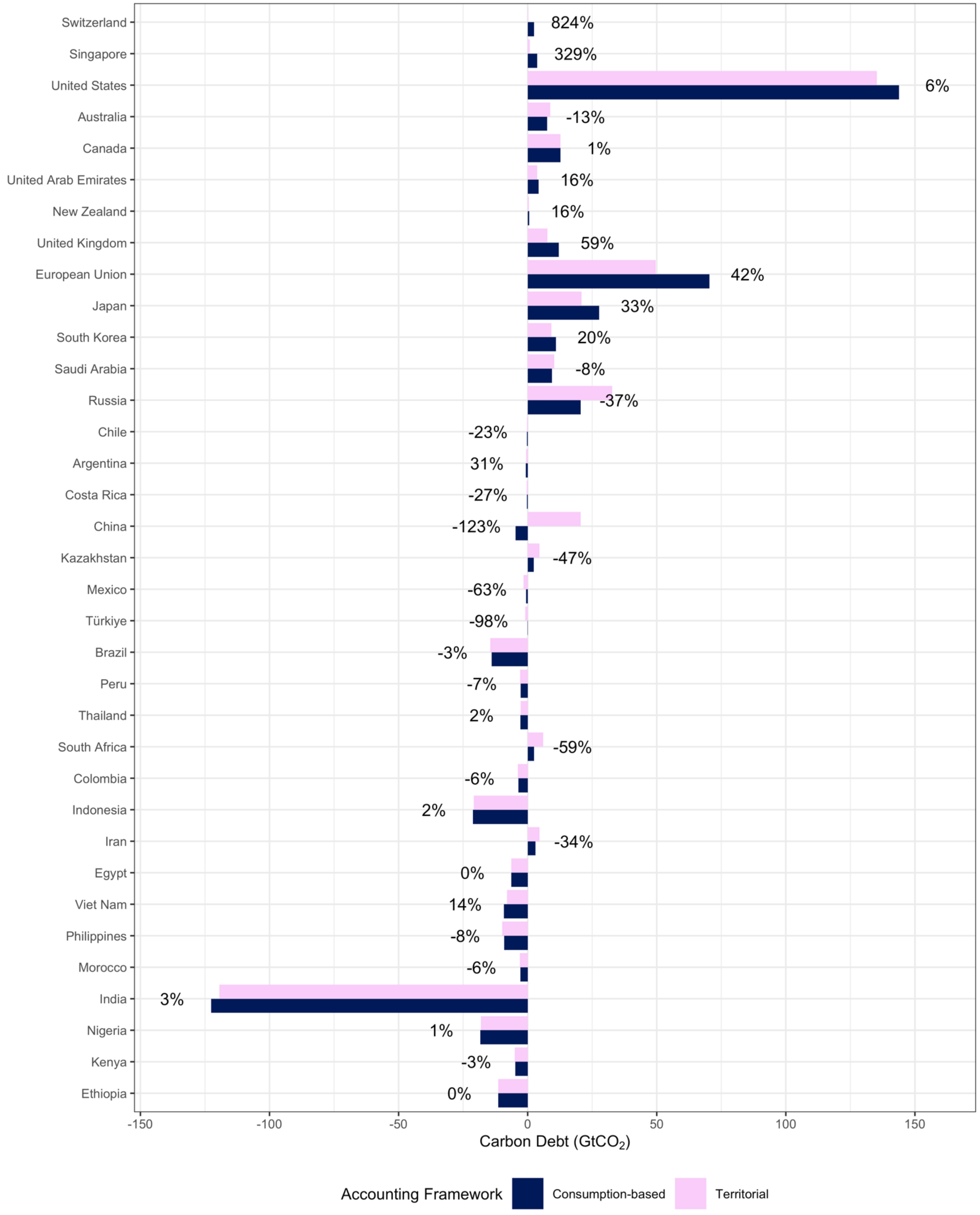

Supplementary Figure 7. **Comparison of carbon debt estimations for different emission accounting frameworks.** Estimated carbon debt using consumption-based (blue) or territorial (pink) CO_2 emission accounting. The effect of a shift from territorial to consumption-based accounting framework is also provided in percent for each individual country. Note that these estimates are not comparable with the carbon debt estimated in the main calculations of this article since the carbon debt estimated in Supplementary Figure 9 are for the period 1990-2021 and that Norway and Gambia are excluded, both of these changes are made due to limited data availability for consumption-based emissions. The estimations are made using equation (1) of the main article. Data from Global Carbon Project[1] are used for territorial as well as consumption-based CO_2 emissions and data from the UN[2] are used for the historical population.

Supplementary Notes

Supplementary Note 1: Allocation methods used in the sensitivity analyses.

This analysis tests how sensitive the results are to the assumed allocation principle for the remaining carbon budget and the principle for redistributing the emission allowances pool. The analysis shows the impact per country in the analysed sample. The analysis covers the allocation principles Grandfathering, Capability, Contraction and Convergence, and Equal cumulative emissions per capita (1990-2070), in comparison with the main case based on equal cumulative emissions per capita 2023-2070 combined with carbon debt since 1990. The sensitivity of the assumed starting year for the carbon debt calculation is also tested. Each analysed case shows the results for a different definition of the excessive carbon claims (equation 2 in the main article) and for the redistribution of the emission allowances pool (equation 6 in the main article). The allocation principles are based on the formulations in van den Berg[3] (Grandfathering, Contraction and Convergence, Equal cumulative emissions per capita) and Robiou du Pont[4] (Capability), see details below. The principles for redistribution of the emission allowances pool are inspired by the same methods with additional reasoning provided below.

The results are provided in Supplementary Figure 4, showing the sensitivity of the assumed starting year for the carbon debt, Supplementary Figure 5, showing the sensitivity of the chosen allocation principle for the remaining carbon budget, and Supplementary Figure 6, showing the sensitivity of the chosen principle for redistributing the emission allowances pool.

Grandfathering

The grandfathering allocation principle is based on current emission levels and would result in the supplementary equation (1) for estimating the excessive carbon claims.

$$Excessive\ carbon\ claims_{Grandfathering} = CE_{country} - CB \cdot \frac{E_{country}(t=2022)}{E_{global}(t=2022)}, \quad (1)$$

where *CE* is the cumulative planned emissions for the country during 2023-2070, *CB* is the global remaining carbon budget for 2023-2070, and *E(t)* are the annual emissions for year *t*.

The emission allowances pool is redistributed proportional to the country's share in the gross total excessive carbon claims among the countries that are assigned an Additional Carbon Accountability, which results in a larger reduction in the Additional Carbon Accountability for countries with large carbon debts and/or large excessive carbon claims. The emission allowances pool for the country, $EAP_{country}$, is defined as in supplementary equation (2):

$$EAP_{country,n} = EAP_n \cdot \frac{ACA_{country,n-1}}{\sum_{i\in\{all\ countries|TECC_i>0\}} ACA_{i,n-1}}, \quad (2)$$

where *EAP* is the global emission allowances pool, and *ACA* is the Additional Carbon Accountability assigned to the country in the previous iteration. Note that the Additional Carbon Accountability is equal to the total excessive carbon claim in the first iteration, $ACA_{country,n=0} = TECC_{country}$.

Equal cumulative emissions per capita

The equal cumulative emissions per capita allocation principle (1990-2070) integrates the concept of carbon debt in the allocation of the remaining budget. Hence, the supplementary equation (3) of the excessive carbon claims replaces the estimated carbon debt and excessive carbon claims of the main scenario.

$$Excessive\ carbon\ claims_{Equal\ cumulative\ emissions\ per\ capita} =$$

$$\sum_{t=1990}^{2022} E_{country}(t) + CE_{country} - \left(\sum_{t=1990}^{2022} E_{global}(t) + CB\right) \cdot \frac{\sum_{t=1990}^{2070} P_{country}(t)}{\sum_{t=1990}^{2070} P_{global}(t)} \quad (3)$$

where *CE* is the cumulative planned emissions for the country during 2023-2070, *CB* is the global remaining carbon budget for 2023-2070, *E(t)* are the annual emissions for year *t*, and *P(t)* is the population in year *t*.

The emission allowances pool is redistributed proportional to the country's share in the future cumulative population among the countries that are assigned an Additional Carbon Accountability. The emission allowances pool for the country, $EAP_{country}$, is defined as in supplementary equation (4):

$$EAP_{country,n} = EAP_n \cdot \frac{\sum_{t=2023}^{2070} P_{country}(t)}{\sum_{i \in \left\{all\ countries | ACA_{i,n-1} > 0\right\}} \sum_{t=2023}^{2070} P_i(t)}, \tag{4}$$

where *EAP* is the global emission allowances pool, and *P(t)* is the population in year *t*.

Contraction and Convergence

The contraction and convergence allocation principle starts in grandfathering and shifts to equal annual emissions per capita by a convergence year, which is assumed to be 2040 in this case. Hence, countries with higher annual emissions per capita than the global average in the starting year will decrease their annual emissions per capita until the convergence year is reached, while countries with lower annual emissions per capita in the starting year will increase their annual emissions per capita until the convergence year is reached. This can be mathematically formulated as in supplementary equation (5).

$$Excessive\ carbon\ claims_{Contraction\ and\ convergence} =$$

$$\sum_{t=2023}^{2070} E_{global}(t) \cdot \left(min\left(\frac{t-2022}{2040-2022}, 1\right) \cdot \frac{P_{country}(t)}{P_{global}(t)} + max\left(1 - \frac{t-2022}{2040-2022}, 0\right) \cdot \frac{E_{country}(2022)}{E_{global}(2022)} \right) \tag{5}$$

where the population projections, *P(t)*, for year *t* is based on UN data. The annual global emissions, $E_{global}(t)$, are estimated based on a linear reduction from the global emission level in 2022 that reaches zero in a year that results in cumulative emissions equal to the remaining carbon budget, *CB*.

Since the allocation principle is a combination of grandfathering and equality, the emission allowances pool is redistributed based on the combination of the country's future cumulative population and its total excessive carbon claims as compared to other countries that are assigned an Additional Carbon Accountability. Hence, the principle accounts for both future population development, the country's carbon debt, and its excessive carbon claims. The emission allowances pool for the country, $EAP_{country}$, is defined as in supplementary equation (6):

$$EAP_{country,n} = EAP_n \cdot \frac{ACA_{country,n-1} \cdot \sum_{t=2023}^{2070} P_{country}(t)}{\sum_{i \in \left\{all\ countries | ACA_{i,n-1} > 0\right\}} ACA_{i,n-1} \cdot \sum_{t=2023}^{2070} P_i(t)}, \tag{6}$$

where *EAP* is the global emission allowances pool, *TECC* is the total excessive carbon claims, and *P(t)* is the population in year *t*.

Capability

The capability allocation principle is based on the projected economic development of the country as compared to the global economic development, as in supplementary equation (7):

$$Excessive\ carbon\ claims_{Capability} = \sum_{t=2023}^{2070} E_{global}(t) \cdot \frac{P_{country}(t)^2 / GDP_{country}(t)}{\sum_{i=\{all\ countries\}} P_i(t)^2 / GDP_i(t)} \tag{7}$$

where the population projections, *P(t)*, for year *t* and the projected economic development in terms of gross domestic product in purchasing power parities, *GDP(t)*, for year *t* are based on the estimates used for the second socioeconomic pathway (SSP2) used by the IPCCs scenarios[5] and downloaded from the SSP Database[6].

The emission allowances pool is allocated proportional to the country's share in the future cumulative gross domestic product among the countries that are assigned an Additional Carbon Accountability. The emission allowances pool for the country, $EAP_{country}$, is defined as in supplementary equation (8):

$$EAP_{country,n} = EAP_n \cdot \frac{\sum_{t=2023}^{2070} GDP_{country}(t)}{\sum_{i \in \left\{all\ countries | ACA_{i,n-1} > 0\right\}} \sum_{t=2023}^{2070} GDP_i(t)}, \tag{8}$$

where *EAP* is the global emission allowances pool, and *GDP(t)* is the gross domestic product in purchasing power parities in year *t*.

Note that the population projections used for Capability therefore slightly diverge from the United Nations' projections. However, we consider it to be important that the population and GDP projections are internally consistent. The annual global emissions, $E_{global}(t)$, are estimated based on a linear reduction from the global emission level in 2022 that reaches zero in a year that results in cumulative emissions equal to the remaining carbon budget, *CB*.

Supplementary References

1. Friedlingstein, P. *et al.* Global Carbon Budget 2023. *Earth Syst. Sci. Data* **15**, 5301–5369 (2023).
2. United Nations, Department of Economic and Social Affairs, Population Division. World Population Prospects: The 2022 Revision. (2022).
3. Van Den Berg, N. J. *et al.* Implications of various effort-sharing approaches for national carbon budgets and emission pathways. *Clim. Change* **162**, 1805–1822 (2020).
4. Robiou Du Pont, Y., Jeffery, M. L., Gütschow, J., Christoff, P. & Meinshausen, M. National contributions for decarbonizing the world economy in line with the G7 agreement. *Environ. Res. Lett.* **11**, 054005 (2016).
5. Intergovernmental Panel on Climate Change. Summary for Policymakers. in *Climate Change 2022: Mitigation of Climate Change. Contribution of Working Group III to the Sixth Assessment Report of the Intergovernmental Panel on Climate Change* (ed. P.R. Shukla, J. Skea, R. Slade, A. Al Khourdajie, R. van Diemen, D. McCollum, M. Pathak, S. Some, P. Vyas, R. Fradera, M. Belkacemi, A. Hasija, G. Lisboa, S. Luz, J. Malley) 3–48 (Cambridge University Press, Cambridge, UK and New York, NY, USA, 2023). doi:10.1017/9781009157926.001.
6. International Institute for Applied Systems Analysis. SSP Database (Shared Socioeconomic Pathways) - Version 2.0. https://tntcat.iiasa.ac.at/SspDb (2018).
